# Datasets on the production routes and the properties of plant powders for manufacturing of green products

**DOI:** 10.1016/j.dib.2024.110787

**Published:** 2024-08-03

**Authors:** Claire Mayer-Laigle, Johnny Beaugrand, Alain Bourmaud, Lena Brionne, Thibault Colinart, Stephane Dervaux, Charlène Fabre, Marie-Joo le Guen, Kolja Konschak, Gabriel Paës, Cécile Sotto, Magalie Weber, Patrice Buche

**Affiliations:** a IATE, University of Montpellier, INRAE, Montpellier SupAgro, 34060 Montpellier, France; b Scion, 49 Sala Street, Rotorua 3010, New Zealand; c INRAE, UR BIA 1268, 44316 Nantes, France; d Université Bretagne Sud, CNRS, IRDL, UMR 6027, Lorient, France; e Université de Reims Champagne Ardenne, INRAE, FARE, UMR A 614, Reims, France; f MIA, Université Paris Saclay, INRAE, AgroParisTech, France

**Keywords:** Fine comminution, Morphological properties, Biochemical composition, Fluorescence spectroscopy, FTIR spectroscopy, Thermogravimetric analysis, Direct sorption measurement, Lignocellulose

## Abstract

The diversity of the plant biomass available on earth makes plants an exceptional resource for replacing fossil resources in green chemistry, bioenergy and biobased materials. For numerous applications, and especially the high-tech ones (building block molecules, high-power bioenergy, additive manufacturing of biobased materials), the macrostructure assemblies of the plant biomass often need to be first broken down into a fine powder. This can be achieved by dry fractionation process combining comminution and sorting steps. The chemical and physical properties of the ground plant powder results both from the process conditions, the histological structure and chemical composition of the raw plant materials. In a forward engineering approach, the quality of the final products can be highly improved by the selection of the right powder (raw materials and production process) for the right application.

This article provides production routes together with physical and chemical characterization of 10 biomass powders from 6 different biomass feedstocks (SP - spirulina, HI - hibiscus, PB - pine bark, HC - hemp Core, RH - rice husk and RHA - rice husk ash). These feedstocks represent a broad range of raw materials properties. For pine bark, hemp core, rice husk and rice husk ash, two grades of powders related to two different particle sizes were produced by two different routes to highlight the impact of the comminution process on the powder properties. The devices used and the process parameters are described. The morphological properties of the powder were quantified using laser diffraction (particle size) and image analysis (shape factor) and qualitatively analyzed with SEM. The specific surface area was determined using gas sorption with BET theory, and the hygroscopic properties were measured using direct vapor sorption. The chemical characterizations were determined with a set of biochemical assays and, complementary, FTIR and fluorescence spectra were recorded to provide fingerprints of samples. The dataset includes tables that summarize the main characteristic descriptors of each analysis as well as the raw data.

The data are registered in the French Research Data Gouv public repository and also stored in the PO2 BaGaTel database using the PO2/TransformON ontology [1]. SPO2Q web tool allows on line querying of the database, which can also be consulted using PO2 manager desktop application [[1], [2], [3]]

Specifications Table

**Table:** 

Subject	Materials processing
Specific subject area	*Production routes of plant powders from various feedstocks with their chemical and physical properties*
Data format	Raw, Analyzed
Type of data	Table, Image, Chart, Graph, Figure, Text, RDF database
Data collection	*Processing of plant materials into powder:* Drying oven (Memmert); cutting mill (SM300, Retsch); impact mill (UPZ, Hosokawa-Alpine); vibrating ball-mill (Sweco, B), sieving machines (Ritec & Rotex,) *Powder characterization:* particle sizer LS 13 320 XR (Beckman and Coulter, shape analyzer QICPIC (SympaTec GmbH), gaz soprtion analyzer ASAP 2460 (Micrometrics,), DVS IGA-sorp-HT system (Hiden Isochema), Ultrapycnometer 1000 (Quantochrome Instrument), colorimeter (Konica Minolta, Inc.), Gas Chromatograph TRACE™ (Thermo Scientific™), elemental analyzer VarioMicro (ELEMENTAR), spectrometer FP-8300 (Jasco instrument), FTIR Tensor 27 (Bruker), SEM JSM-IT500HR (JEOL)
Data source location	***INRAE*** in - PLANET-IATE, 1208. Agropolymers and Emerging Technologies Facility, *Montpellier FR-34060, France*https://doi.org/10.15454/1.5572338990609338E12 - BIA 1268 Biopolymères Interactions Assemblages, Nantes FR-44316 - Fractionation of AgroResources and Environment (FARE) laboratory, Reims, France
	**SCION**, New Zealand Forest Institute, 49 Sala Street, *Rotorua* NZ-3010**Univ. Bretagne Sud**, UMR CNRS 6027, IRDL, Lorient, France
Data accessibility	Repository name: Research data gouv (https://entrepot.recherche.data.gouv.fr/) Data identification number: *(or DOI or persistent identifier)* Dataset 1: 10.57745/LNF4RF Dataset 2: 10.57745/CVN0MI Dataset 3: 10.57745/1SVOBA Direct URL to data: Dataset 1: https://doi.org/10.57745/LNF4RF Dataset 2: https://doi.org/10.57745/CVN0MI Dataset 3: https://doi.org/10.57745/1SVOBA Instructions for accessing these data: open access. Videos explaining how to use PO2 are available in ***Dataset 1*** (named *'Consultation of a PO2 project using PO2 Manager.mp4*' and *'SPARQL query execution with SPO2Q.mp4*')

## 1 Value of the Data

• Plant biomass can replace petroleum-based materials in many applications including bioenergy, materials and green chemistry [[4], [5], [6], [7]]. According to the nature of the starting biomass material and the comminution process, variability in particle characteristics can be introduced [8,9]. The properties of end products and their quality are directly related to those of the plant powder. These data, obtained from various biomass feedstocks using different types of mills, may guide the choice of starting feedstock and processing technologies according to the targeted applications, enabling the better exploitation of biomass potential as petroleum replacements.
• Given the wide range of biomass species and diversity of the data compiled, this dataset can provide valuable benefits to scientists and companies engaged in the production of plant and wood-based powders for many applications, as well as mill suppliers. It is particularly relevant to those involved in the fields of plant and wood product valorization who are exploring new uses for plant biomass by-products. This information can facilitate the development of innovative products and more efficient technologies for the comminution of plant biomass.
• The dataset can be used to compare different characterizations of plant powders obtained from various measurements, such as the analysis of shape and size factors using SEM images. It can also be used to correlate certain analyses in order to explain the relationship between different properties. For example, correlations between biochemical data and data from fluorescence spectroscopy and FTIR could be investigated, as well as those of specific surface areas with water sorption responses.
• This dataset complements existing data (dataset or online available database) [9,10]. By combining all of this data, it might possible to feed machine learning tools to develop models that can predict the grinding behaviour of different biomass materials and the properties of the resulting powders.
• By providing a comprehensive set of features for plant biomass powders and the technologies to produce them, this dataset can also pave the way for ground-breaking innovations in the realm of green products. By comparing these characteristics to those of currently non-renewable (or unsustainable) powders, it becomes possible to substitute these powders with sustainable alternatives and transform consumption patterns.

## 2 Background

Plant biomass is a substantial and diverse resource, housing valuable components intricately woven into a complex structure, offering a sustainable alternative to fossil-based resources. To unlock them, a fractionation process is required, which affects not only the physical but also the chemical properties of the feedstock, potentially impacting the functionalities of the resulting powder. These functionalities are crucial attributes for the end-products, making the powder valuable for energy vectors, smart materials, and cosmetic/biomedical applications. These datasets were generated within the framework of the SMARTPOP project (MSCA-H2020-893040), where the diversity of plant powders was explored for biocomposite materials intended for 3D printing. Six biomass species with contrasting physical and chemical properties were selected, and powder preparation was carried out through various technological pathways, resulting in ten powders presenting two targeted particle sizes. The powders were extensively characterized. While we have used relevant data for the project, our aim is to share the entire dataset with the scientific community, providing a foundation for other applications in related fields.

## 3 Data Description

Data, materials, and methods are stored in three datasets. The different powders used have been labelled as described below. To provide a range of sizes, the median particle size, measured without applying ultrasonic treatment is also provided (see the Materials and Methods section). The labels of the powders are also reported in Table 1.1 of Dataset 1, which lists the feedstocks.

• **RH-1**: Coarse rice husk powder (d_50_ = 433.10 µm)
• **RH-2**: Fine rice husk powder (d_50_= 29.61 µm)
• **RHA-1**: Coarse rice husk ash powder (d_50_ = 31.23 µm)
• **RHA-2**: Fine rice husk ash powder (d_50_ = 13.05 µm)
• **HC-1**: Coarse hemp core powder (d_50_ = 160.60 µm)
• **HC-2**: Fine hemp core powder (d_50_ = 23.27 µm)
• **PB-1**: Coarse pine bark powder (d_50_ = 47.30 µm)
• **PB-2**: Fine pine bark powder (d_50_ = 17.35 µm)
• **HI**: Fine hibiscus powder (d_50_ = 41.76 µm)
• **SP**: Fine spirulina powder (d_50_ = 11.32 µm)
• ***Dataset 1 - M&M***, gathered all the information relative to the Materials and methods.

This dataset includes three files describing the origin of the feedstocks (Table 1.1: *feedstocks provenance*), the processing itineraries for producing the ten powders (Table 1.2: *steps list*) and the controlled parameters associated with steps for each itinerary (Table 1.3 *steps and controlled parameters list*), respectively, as well as 11 text files, each corresponding to one method of characterization.

In *Table 1.1 (Feedstock*s), the third column corresponds to the reference of the powders, relative to production routes (or itinerary), while the columns five and nine contain metadata describing the type of the feedstocks and their origin.

In *Table 1.2 (Steps list*), each processing route is described by a set of unit operations and associated with a unique numerical identifier (column ʻitinerary labelʼ). Each unit operation of a given itinerary is described on a separate line and associated with a unique alphanumerical identifier (including the itinerary number, step number and type of action performed on the biomass). The numerical value associated with the step_1 column indicates the temporal order (e.g., step 1.0 is before step 1.1).

Table 1.3 (*Steps and Controlled Parameters List*) compiles information about the parameters associated with each processing step. This includes references to the materials used (found in the ʻStep Materialsʼ column), the types of parameters measured, along with their corresponding values and units (provided in the ʻMaterial Parameter,ʼ ʻValue Origin Material Parameter,ʼ and ʻUnit Origin Material Parameterʼ columns, respectively.

The dataset also includes the SPARQL queries used to generate the tables from the online tools SPO2Q. Queries names and table names have the same name, only the extension differs replacing the string ‘tsv’ by ‘sparql’. By example ‘*Table 1.1 feedstocks provenance.sparql’ is the name of the query associated with table ‘Table 1.1 feedstocks provenance.tsv’.* Furthermore, the dataset also includes two videos: one demonstrating the execution of SPARQL queries stored in ***datasets 1*** and ***2*** (‘*SPARQL query execution with SPO2Q*’) [15], and another explaining the installation of PO2 Manager and how to access the entire experimental project using this desktop application (‘*Consultation of a PO2 project using PO2 Manager’*) [16].

The text files corresponding to the materials and methods are named based on the characteristic parameters measured as in the following section: Experimental design, Materials, and Methods.

• ***Dataset 2 – Raw and calculated data***

**Fig. 1:**
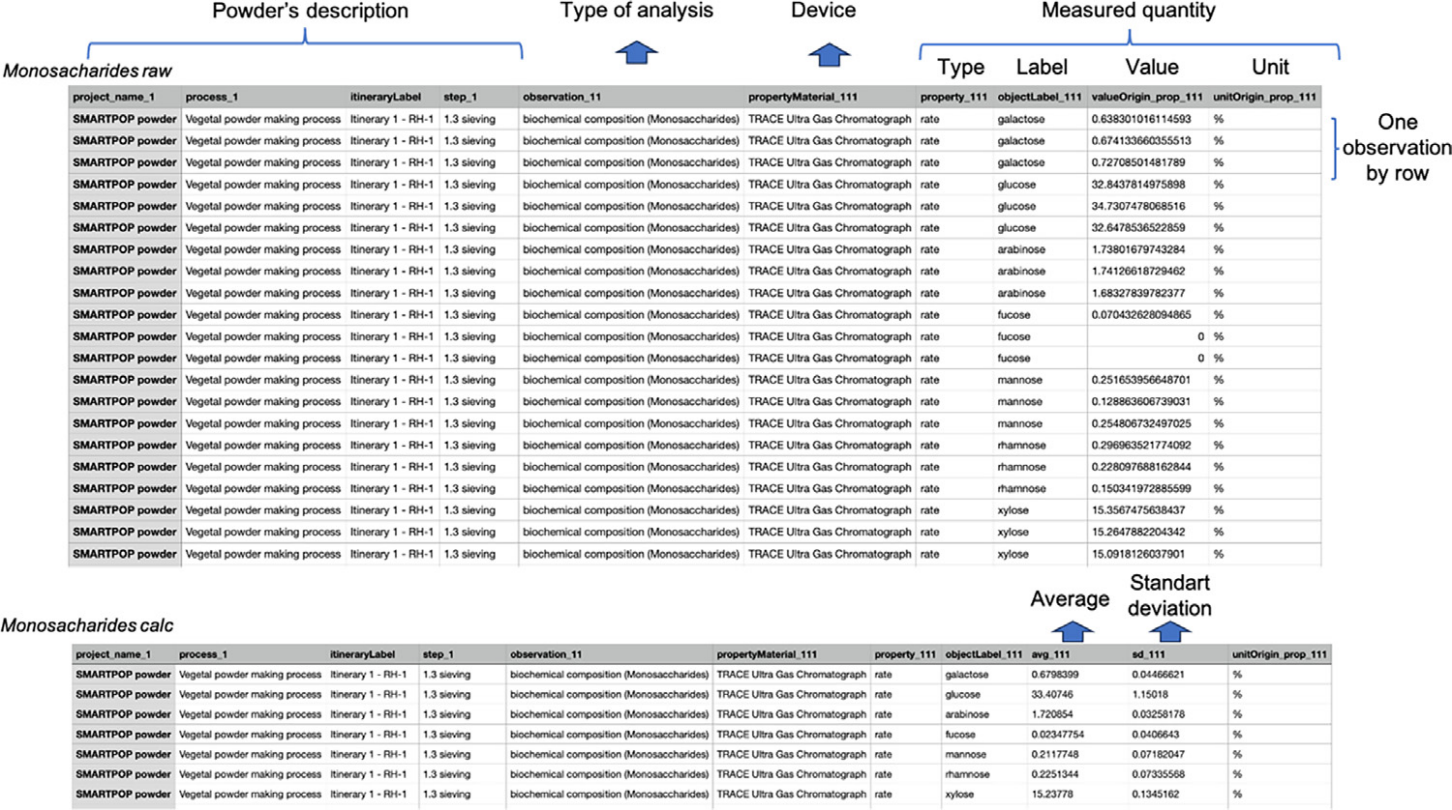
Example table for raw and calculated data obtained in the analysis of monosaccharides (biochemical composition).

For each analysis performed on the powders, this dataset includes a file including a table with the raw data (*analysis* raw), and in cases where multiple observations of the same parameter were made, there is also a file including a calculated table (*analysis* calc) that includes the average and standard deviation values for the several observations. Fig. 1 shows an example of a table for both raw and calculated data obtained during the analysis of biochemical composition, along with explanations of the different column labels.

For all of these tables, the first four columns (project name, process, itinerary label, and step_1) contain the primary information regarding the powders, the production itinerary, as well as the last processing step. The following column (observation) corresponds to the types of analyses performed. The column *‘property materials*ʼ describes the type of equipment used to perform the analysis. Finally, the last columns are related to the different measured characteristics (type of quantity, name of the quantity, value, and units of measurement) in the table.

For the raw data tables, each observation of the same value is reported in a different row, as illustrated in Fig. 1. For the calculated data, the averages and standard deviations (or deciles for particle size distributions) of the different observations of the same quantity are calculated and reported in the columns *‘avg_111ʼ* and *‘sd_111ʼ*.

The different quantities measured and the corresponding tables for each analysis are reported below. The dataset also includes the SPARQL queries used to generate the tables from the online tools SPO2Q. Queries and table have the same name, only the extension differs replacing the string ‘tsv’ by ‘sparql’. By example ‘*Table 2.1 monosaccharides raw.sparql’* is the name of the query associated with table *‘Table 2.1 monosaccharides raw.tsv’ .*

### 3.1 Biochemical composition

- The monosaccharides concentration for: galactose, glucose, arabinose, fructose mannose, rhamnose, xylose and uronic acid (% w/w) (tables Table 2.1 monosaccharides raw and Table 2.2 monosaccharides calc)

The lignin (*Table 2.3 lignin raw and Table 2.4 Calc*), ash (*Table 2.5 biochemical ash rate raw)*, protein content (*Table 2.6 protein rate raw and Table 2.7 protein rate calc*)
*Color* (Table 2.8 colorimetry raw and Table 2.9 colorimetry calc): attributes (L*, a*, b*) in the CIE LAB chromatic space, where L* is an indicator of clarity, a* represents a value on the green-red axis, and b* represents a value on the blue-yellow axis.
*FTIR Spectra* (Table 2.10 Fourier transform infrared raw): Main peaks of absorbance with their wavenumber, corresponding absorbance, intensity and width.
*BET Specific surface* (Table 2.11 specific surface area raw and Table 2.12 specific surface area calc) with

- The measured specific surface area,
- The BET constant (also known as constant C), which is proportional to the fraction of solid surface not covered by nitrogen molecules under conditions where adsorption is sufficient to theoretically cover the solid surface with a monolayer,
- The correlation coefficient 'R-squared,' representing the discrepancy between the measured values and the regression performed for calculating the specific surface area.

*Spectrofluorimetry* (Table 2.13 spectrofluorimetry raw): Main peaks of absorbance (ordered by excitation wavelength) with the corresponding excitation wavelength, emission wavelength, and peak intensity.

*Particle size* (Table 2.14 Particle size calc): the volumetric D10, D50, D90 representing the 10th, median, and 90th percentiles of the distribution, the span ((D90-D10)/D50), and the specific surface area for measurements carried out with or without previous desagglomeration using an ultrasonic treatment to disperse particles that may aggregate during the milling step.

*Particle shape* (*Table 2.15 Particle shape length calc*): the 10th, 15th, median, 85th and 90th percentiles of the length (Ferret Max); (*Table 2.16 Particle shape diameter calc*) diameter (Feret Min); and (*Table 2.17 Particle shape aspect ratio calc*) aspect Ratio (length/diameter).

*Thermogravimetric analysis*(Table 2.18 Thermogravimetric ash and water content and Table 2.19 Thermogravimetric main transition temperatures) with the ash rate, the water content and the main transition temperatures.

*True density:* (Table 2.20 true density raw and Table 2.21 true density calc): with the volume, mass and density measured.

*Water sorption properties:* (Table 2.22 Direct Vaper Sorption raw) For each humidity level, the corresponding mass is reported. The corresponding mass gain can be calculated from the dry mass (at 0 % humidity).

### 3.2 Dataset 3 – spectra and images (organized in 11 folders)

**Fig. 2:**
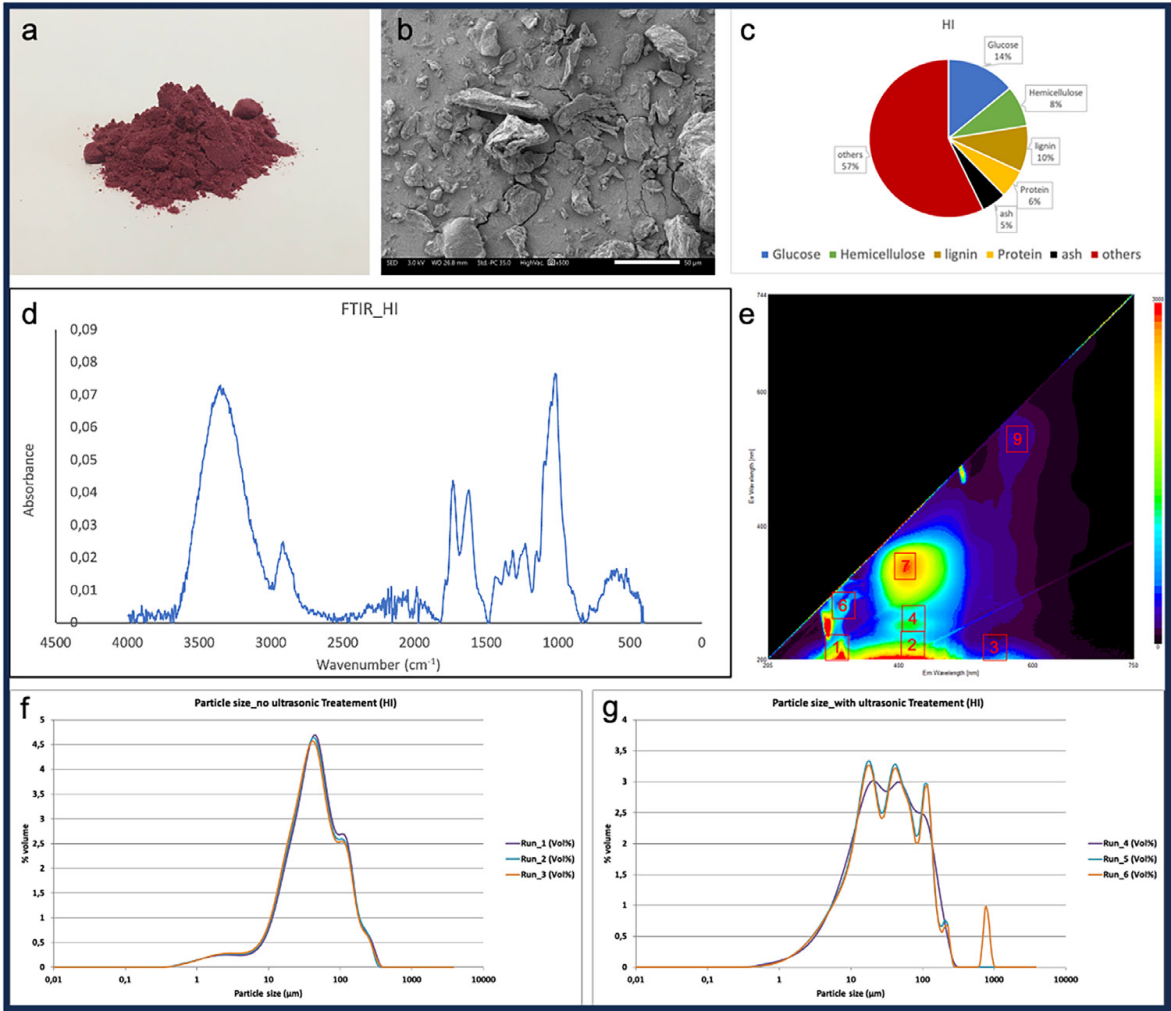
Example of the various characterization images and spectra available in *Dataset 3* for each type of powder. Optical and SEM images of the ground powder (a and b); chemical composition (c); FTIR and fluorescence spectra (d and e); particle size measurements without and with ultrasonic treatment (f and g).

***Dataset 3*** includes photographs of the powders, SEM images, as well as various spectra and graphs related to other measured properties, such as particle size, FTIR spectra, spectrofluorimetry spectra, and thermogravimetric measurements. Fig. 2 provides an example of the various graphs included in this dataset for the powder HI (Hibiscus powder). These data are available in .txt, .csv files or image formats. All the files are organized into folders, with one folder for each powder. Filenames begin by the abbreviation associated with the powder. By example HI_biochemistry.png is an image containing information about biochemical constituents associated with Hibiscus powder. The dataset also includes an additional folder containing a general overview of the biochemical composition of the ten powders (table) and several graphs (bar and pie charts) for comparing global and monosaccharide contents.

## 4 Experimental Design, Materials and Methods

### 4.1 Materials origin

The six biomass feedstocks used to generate the ten powders in the dataset were either kindly provided by French companies or purchased from a local store. All the origin information is gathered in *Table 1.1* within the dataset, which is named *feedstocks*.

### 4.2 Powder processing

Information about the devices and parameters used for processing each feedstock to produce the ten powders is documented in the Table *step List*. Various technological steps were taken for this purpose.

*Drying:* Feedstocks are dried before the intermediate and fine milling steps, as outlined in Table 1.2, utilizing a conventional drying oven (Memmert, Germany) set at 60 °C for a duration generally of 24 h, or as specified in Table 1.2 (*dataset 1*).

*Milling:* Three different milling steps may have been applied to the different feedstocks

(i) *Coarse milling*: performed using an SM 300 cutting mill (Retsch, Germany) operating at 3000 rpm and equipped with a 2 mm grid.
(ii) *Intermediate milling:* using the UPZ impact mill (Hosokawa-Alpine, Germany) operating at 18,000 rpm with a 0.3 mm grid, and optionally at 0.1 mm (in the case of spirulina).
(iii) *Fine milling:* conducted in batches using a vibrating ball mill (Sweco, Belgium) with 25 kg of 12 mm diameter balls and 25 kg of cylindrical bodies measuring 12 × 12 mm as grinding media, for processing 1 kg of plant materials. The mill operates at 25 Hz for a duration ranging from 5 to 210 min.

*Sieving:* Sieving steps were performed to generate the samples RH1, RHA1, and RHA2, as explained below

• The sample RH1 was obtained by sieving the ground rice husk obtained after the coarse milling step using a vibrating sieving machine (Ritec, France). The sample corresponds to the fraction between the 0.2- and 0.4-mm grids.
• For samples RHA 1 and RHA 2, sieving was performed using a rotating sieving machine (Rotex, France) equipped with a 0.8 mm grid to remove larger blocks of materials prior to fine milling.

### 4.3 Powder characterizations

The following characterization was carried out for all powders produced following the different protocols described below. Note that all the protocols are also gathered in the *dataset 1 Materials and Methods*.

### 4.4 Biochemical composition

• *Monosaccharides*. Total cell wall monosaccharides were measured as previously by Lahaye et al. (2020) [11]. Briefly, 5 mg of samples were acid hydrolysed with strong sulfuric acid, and the resulting liberated neutral sugars were derivated into alditol-acetates and analyzed on a TG-225 GC Column (30 × 0.32 mm ID) using TRACE™ Ultra Gas Chromatograph (Thermo Scientific™; temperature 205 °C, carrier gas H2). Uronic acids were quantified according to the metahydroxy-diphenyl colorimetric method [12]. All measurements were performed in triplicates using standard monosaccharide solutions and inositol as internal standard for calibration.
• *Lignin*. The lignin content was quantified by spectrophotometry following the acetyl bromide method [13] on samples weighting approximately 20 mg each. The chemicals were laboratory grade from Sigma Aldrich, and the analyses were performed with four independent assays. The lignin content is expressed as the percentage of the dry weight.
• *Protein*. The total C and N contents of the samples were determined on cryogrinded powders via the Dumas method using an elemental analyzer (VarioMicro, ELEMENTAR). Protein contents were determined from N contents multiplied by a 5.7 coefficient usually applied for non-reserve proteins. Experiments were run in triplicate.
• *Ash.* Approximately 2 gs of powder underwent an initial drying process in an oven at 130 °C for 90 min to determine its dry mass. Subsequently, the sample was heated at 900 °C for 2 h, followed by cooling in a desiccator until it reached room temperature and then weighed (referred to as the residual mass). The ash content was calculated as the ratio of the residual mass to the dry mass. Experiments were conducted in triplicate and the results were averaged.

The ***dataset 2*** includes the mean value for each entity (Cell wall monosaccharides, lignin, protein, and ash content) as well as the raw data corresponding to each experiment. Graphs comparing the overall composition of the different powders are also provided in ***dataset 3***.

*Color:* The color attributes of the different powders were measured using a colorimeter (Konica Minolta, Inc., Tokyo) with the L*, a*, b* color system. To ensure the exclusive measurement of the powder's color, a thick layer of powder was placed inside a black-bottomed container equipped with a lid featuring an aperture matching the size of the colorimeter's cover. The measurements were conducted in triplicate and then averaged. The L*-value represents the brightness of the sample on a scale from 0 (black) to 100 (white), and the b*-value represents the blue-yellow axis, ranging from −60 (blue) to 60 (yellow).

*FTIR Spectra*: FTIR spectra were recorded using the ATR module of the FTIR Tensor 27 (Bruker, USA). Prior to the measurements, the powders were dried in an oven at 60 °C for 24 h. For each powder, three measurements were performed with 64 scans each. Baseline correction, normalization performed on the three replicates, and the average were calculated. The main absorbance peaks and their corresponding absorbance values were extracted from the average spectra using the OPUS software and reported in ***dataset 2***. The corresponding spectra for each powder are also included in ***dataset 3*** in separate Excel files.

*Specific surface*: The specific surface area was determined through B.E.T measurements using nitrogen as the gas carrier, following the method employed by Rajaonarivony et al. [14].. In addition to the specific surface area, the dataset also includes the value measured for the constant C, which is proportional to the fraction of solid surface not covered by nitrogen molecules, even though sufficient adsorption has occurred to theoretically cover it with a monolayer. Additionally, the correlation coefficient R^2 represents the discrepancy between the measured values and the regression performed for the calculation of the specific surface area are also reported. These data are reported in the ***dataset 2,*** in the tables BET specific surface raw and calc.

*Particle size:* The particle size distributions and the primary granulometric indicators were measured using laser diffraction with the LS 13 320 XR particle sizer equipped with the universal liquid module combined with a sonicator (Beckman & Coulter).

In the first protocol (protocol 1), the particle size was measured as is (raw particle size measurement). In the second protocol (protocol 2), the powder was first deagglomerated using ultrasonic treatment to determine the real surface area of the powder after the comminution process, which is associated with its potential reactivity.

• Protocol 1: Raw Particle size measurement

Measurements were carried out with ethanol as a solvent. First, a debubbling phase was applied to remove any bubbles that the filling of the cuvette may have induced. Then, the particle size analyzer was loaded with the powder until it reached 2 % of charge. The suspension was then stirred at 45 % of the maximum pump speed with 2 min of sonication at the maximum power of the particle size analyzer probe (100 %). Measurement was performed three times.

• Protocol 2: Particle size measurement after desagglomeration by ultrasonic treatment

38 mm^3^ of powder was dispersed in 50 ml of 96 % v/v ethanol. Ultrasonic treatment was then applied using an ultrasonic probe (QSonica, US) at 70 W of amplitude for three minutes. The particle size analyzer was loaded with the suspension until it reached 2 % of the sample charge after the debubbling phase. The suspension was then stirred at 45 % of the maximum pump speed, and a complementary ultrasonic treatment was applied for 2 min using the internal sonicator of the particle size analyzer at the maximum power of the probe (100 %). Six measurements of 30 s each were performed. As bubbles were still present in the first 3 measurements, the mean particle size distribution was calculated from the last three measurements.

The main indicators, d10, d50, and d90, as well as specific surface area and SPAN, were extracted from the particle size distribution and are reported in the ***dataset 2***. The full particle size distributions are compiled in ***dataset 3*** as .csv files and images.

*Particle shape:* The morphology of the plant powders was investigated using a dynamic image analyzer (QICPIC, SympaTec GmbH, Germany) equipped with the M5 lens, allowing the detection of particles ranging from 1.8 µm to a maximum of 3755 µm and the determination of various shape factors within the range of 16 µm to 1252 µm. Briefly, 150 mg of each powder was weighed and suspended in 20 ml of ethanol and 20 ml of distilled, filtered water. To prevent particle agglomeration, the suspension was stirred using an ultrasonic device. Subsequently, an additional 960 ml of distilled, filtered water was added, and the suspension was continuously stirred with a magnetic stirring rod. A pump was employed to circulate the suspension through the QICPIC system for analysis, and it was finally returned to the measuring beaker in a closed circuit. Depending on the sample, between 340,000 and 2.8 million particles were analyzed.

The PAQXOS software (SympaTec GmbH, Germany) was utilized to determine the minimal and maximal Ferret diameters, which were associated with the width (D) and length (L) of the particle, respectively. Additionally, the aspect ratio was calculated as the ratio of the maximal to the minimal Feret diameter. Feret diameters do not represent traditional diameters but instead measure the distance between two tangents to the particle's contour in a well-defined orientation. The software calculates both the maximal and minimal Ferret diameters for each particle, considering all possible orientations (0° to 180°). In the case of irregularly shaped particles, the Ferret diameter can vary significantly more than with regularly shaped particles. Therefore, the maximum and minimum Ferret diameters can be significantly larger and smaller, respectively, than the diameter of the equivalent circle.

The ***dataset 2*** includes the values of the 10th, 15th 50th, 85th and 90th percentiles of the minimal and maximal Ferret diameters, as well as the aspect ratio calculated from them.

*SEM pictures:* The plant powders from each milling itinerary were initially coated with a thin layer of gold using the Edwards Sputter Coater from the Atlas Copco Group in Sweden. Subsequently, they were observed using a scanning electron microscope (Jeol JSM-IT500HR, JEOL Ltd., Japan). Images were captured at magnifications of 25×, 100×, 500×, 1000×, and 2000× for each sample, resulting in a total of 5 images per sample. All the images are gathered in ***dataset 3*** and organized into one folder per powder.

*Spectrofluorimetry:* 40 mg of powder were placed in a powder holder with a quartz window and the 3D fluorescence spectra of the powders were measured in a Jasco FP-8300 instrument (Lisses, France). Acquisition parameters were as follows: range/precision for excitation and emission were 200–744 nm/2 nm and 205–750 nm/1 nm, respectively; The excitation and emission bandwidths were both 2.5 nm; scan speed was 1000 nm/min; gain value was 500 V. Spectra acquisition was performed using Jasco Spectra Manager software to obtain 3D and 2D spectra (corresponding to the spectrum recorded for the maximal emission wavelength).

The ***dataset 3*** contains the raw data corresponding to the 2D and 3D spectra as csv files, as well as the corresponding images.

*Thermo-Gravimetric Analysis* (TGA) were performed using a TGA2050 (TA Instruments – Waters SAS, France). Around 2 mg to 5 mg of each sample were placed in a platinum crucible and heated at 10 °C.min^−1^ from ambient temperature to 700 °C under nitrogen atmosphere. The weight loss was recorded during the heating and plotted as a function of temperature.

From the derivative curve, the temperatures corresponding to the main mass losses were determined using the peak maxima, along with the initial water content and the percentage of residual ash at the end of the tests. ***Dataset 2*** contains these data. The full spectra are saved as text files with the corresponding images in PDF in the ***dataset 3***.

*True density:* was measured using an Ultrapycnometer 1000 (Quantochrome Instrument) with nitrogen as the gas carrier. Few grams of powder were initially weighed in the measurement cell. Subsequently, a vacuum degassing cycle was applied three times to remove any air present in the cell. Three runs were then performed on the powder to determine its volume, and the mean value of these three runs was calculated. The true density was determined by calculating the ratio of the mass to the average volume. Measurements were repeated three times. The data are gathered in the ***dataset 2***.

*Water sorption properties:* Water sorption properties were determined through adsorption and desorption isotherms measured with the DVS equipment IGA-sorp-HT system (Hiden Isochema, Warrington, UK). A quantity of 15–30 mg of the product was placed inside a stainless-steel mesh basket, suspended from a microbalance with a resolution of 10^–7 kg. The sample was then placed inside a chamber with controlled humidity and temperature. Prior to the adsorption experiment, the sample was dried under a flow of dry nitrogen at 105 °C for 1 h (with a flow rate of 250 mL/min) until it stabilized. The dry mass of the sample was recorded after setting the temperature to 23 °C under a flow of dry nitrogen. Subsequently, the sorption/desorption sequence was programmed as follows: increase from 0 % to 90 % RH at 10 % RH steps, measurement at 95 % RH, and decrease to 0 % RH at 10 % RH steps. For each RH step, the sample mass was continuously measured until equilibrium with the surrounding air was reached, which was indicated by a mass variation of less than 0.1 µg/min over a 1-hour period. ***Dataset 2*** includes the raw data corresponding to the mass measured for each%RH step (Table *Direct Vaper Sorption raw).*

### 4.5 Experimental data structuration

The data are registered in the French Research Data Gouv public repository and also stored in the PO2 BaGaTel database using the PO2/TransformON ontology [1]. SPO2Q web tool allows on line querying of the database with SPARQL queries registered in ***datasets 1 and 2***. One may also consult the experimental data project using PO2 manager desktop application. Two video presentations are available ***in dataset 1*** to show (1) how SPARQL queries stored in ***datasets 1 and 2*** can be executed [15], (2) how to install PO2 Manager and consult the entire experimental project using this desktop application [16].

## Limitations

The datasets are not entirely complete. Some data are missing for certain powders, as described and explained below.

***Optical and SEM*** (Scanning Electron Microscope) images could not be recorded for the HC-1 powder due to its unavailability at the site where these measurements were performed.

*Specific surface measurements* using BET methods for the PB-1 route powder did not yield reliable results due to difficulties in obtaining a proper sorption isotherm. This was attributed to the complex chemical surface composition of pine bark powder, which complicates the application of the B.E.T. theory to such powders.

***The water sorption properties*** have only been recorded using DVS (Dynamic Vapor Sorption) for four powders (SP, PB-2, HI, and HC-2). Consequently, the DVS measurements are missing for the powders HC-1, RH-1, RH-2, RHA-1, RHA-2, and PB-1. We had limited access to the equipment, and characterization priority was given to the powders that were subsequently used in composite – however we think it is still of value to report the other ones.

## Ethics Statement

The authors have read and follow the ethical requirements for publication in Data in Brief and confirm that the current work does not involve human subjects, animal experiments, or any data collected from social media platforms.

## CRediT authorship contribution statement

**Claire Mayer-Laigle:** Conceptualization, Methodology, Validation, Investigation, Data curation, Writing – original draft, Visualization, Supervision, Project administration, Funding acquisition. **Johnny Beaugrand:** Methodology, Validation, Investigation, Data curation, Writing – review & editing. **Alain Bourmaud:** Methodology, Validation, Investigation, Data curation, Writing – review & editing. **Lena Brionne:** Validation, Investigation, Data curation, Writing – review & editing. **Thibault Colinart:** Methodology, Validation, Investigation, Data curation, Writing – review & editing. **Stephane Dervaux:** Software. **Charlène Fabre:** Methodology, Validation, Investigation, Data curation. **Marie-Joo le Guen:** Methodology, Validation, Investigation, Data curation, Writing – review & editing. **Kolja Konschak:** Methodology, Validation, Investigation, Data curation. **Gabriel Paës:** Methodology, Validation, Investigation, Data curation, Writing – review & editing. **Cécile Sotto:** Validation, Investigation, Data curation. **Magalie Weber:** Data curation, Writing – review & editing. **Patrice Buche:** Conceptualization, Methodology, Validation, Investigation, Data curation, Writing – original draft, Visualization, Supervision.

## Data Availability

Production routes and properties of various plant powders intended for the manufacture of green products: raw and calculated analytical data (Original data) (Research Data Gouv) Production routes and properties of various plant powders intended for the manufacture of green products: Spectra and Images (Original data) (Research Data Gouv) Production routes and properties of various plant powders intended for the manufacture of green products: materials and methods (Original data) (Research Data Gouv)
